# Diagnostic utility of CT for small bowel obstruction: Systematic review and meta-analysis

**DOI:** 10.1371/journal.pone.0226740

**Published:** 2019-12-30

**Authors:** Zhengyan Li, Ling Zhang, Xijiao Liu, Fang Yuan, Bin Song

**Affiliations:** 1 Division of Radiology, West China Hospital of Sichuan University, Chengdu, Sichuan, China; 2 Division of Nephrology, West China Hospital of Sichuan University, Chengdu, Sichuan, China; Wayne State University, UNITED STATES

## Abstract

**Background:**

To perform a systematic review and meta-analysis evaluating the diagnostic performance of computed tomography (CT) for small bowel obstruction (SBO), including diagnostic accuracy, ischemia, predicting surgical intervention, etiology and transition point.

**Methods:**

PubMed/MEDLINE and related databases were searched for research articles published from their inception through August 2018. Findings were pooled using bivariate random-effects and summary receiver operating characteristic curve models. Meta-regression and subgroup analyses were performed to evaluate whether publication year, patient age, enhanced CT, slice thickness and pathogenesis affected classification accuracy.

**Results:**

In total, 45 studies with a total of 4004 patients were included in the analysis. The pooled sensitivity and specificity of CT for SBO were 91% (95% confidence interval [CI]: 84%, 95%) and 89% (95% CI: 81%, 94%), respectively, and there were no differences in the subgroup analyses of age, publication year, enhanced CT and slice thickness. For ischemia, the pooled sensitivity and specificity was 82% (95% CI: 67%, 91%) and 92% (95% CI: 86%, 95%), respectively. No difference was found between enhanced and unenhanced CT based on subgroup analysis; however, high sensitivity was found in adhesive SBO compared with routine causes (96% vs. 78%, P = 0.03). The pooled sensitivity and specificity for predicting surgical intervention were 87% and 73%, respectively. The accuracy for etiology of adhesions, hernia and tumor was 95%, 70% and 82%, respectively. In addition, the pooled sensitivity and specificity for transition point was 92% and 77%, respectively.

**Conclusions:**

CT has considerable accuracy in diagnosis of SBO, ischemia, predicting surgical intervention, etiology and transition point.

## Introduction

Small bowel obstruction (SBO) is a common emergency diagnosis based on clinical signs and radiographic findings and is estimated to account for 2% of all patients with abdominal pain and 12–16% of hospital admissions in the United States [1, 2]. Although most SBO patients are treated successfully with nasogastric tube decompression, the mortality of SBO increases to 25% if bowel ischemia is present with delayed surgical management [3]. A challenge in the clinical management is that clinical presentation, physical examination findings, and laboratory tests are insufficient to make an accurate diagnosis of SBO, but the management has changed considerably since then due to advancements in imaging technology, such as computed tomography (CT) [3, 4].

There is a consensus regarding the use of CT in the evaluation of patients with SBO [5], and many studies reported that CT is a helpful tool to diagnose SBO, identify causes and the transition point, and most importantly, detect ischemia, which requires prompt surgical intervention [6–18]. However, CT findings about such points exhibit variable diagnostic performance. For example, the sensitivity of CT for diagnosis of SBO varied from 50% to 100%, and the specificity values varied from 57% to 100% [1, 8, 19–22]. Furthermore, many studies reported that CT had high sensitivity and specificity for ischemia of SBO [7, 8, 12, 23–25], but a prospective study [26] showed a poor sensitivity of 15% for ischemia. In addition, several studies showed CT could predict surgical intervention for patients with SBO [9, 11, 13, 27], but a recent study including 108 cases found no correlation between CT findings and surgical intervention [28].

Given the inconsistency in the existing literature, we performed a quantitative meta-analysis using bivariate random-effects and summary receiver operating characteristic models to evaluate the diagnostic performance of CT for SBO, including diagnostic accuracy, ischemia, predicting surgical intervention, etiology and transition point.

## Materials and methods

No financial support was received for this research. We performed this systematic review using the guidelines proposed by the Cochrane Collaboration in the Cochrane Handbook for Systematic Reviews of Diagnostic Test Accuracy (http://srdta.cochrane.org/handbook-dta-reviews). The protocol was registered on PROSPERO (CRD42015024658) [29].

### Study selection criteria

1. Participants
          This review focused on patients with suspected diagnosis of SBO.
2. Index tests
      CT was the test under evaluation.
3. Target conditions
      The target condition of this review was to evaluate the diagnostic performance of CT for SBO, including diagnostic accuracy, ischemia, predicting surgical intervention, etiology and transition point.
4. Reference standards
      The primary reference standard was surgical findings in patients who received surgical intervention. Alternatively, clinical findings and enteroclysis were the reference standards if the patients did not receive any surgical intervention.

### Search methods for identification of studies

#### 1). Study selection

We used the Cochrane risk of bias tool and the PRISMA (Preferred Reporting Items for Systematic Reviews and Meta-Analyses) statement methodology [30] to report systematic reviews and perform the meta-analysis **(S1 Table)**. Two independent reviewers (Z.L. and L.Z.) conducted a search of Medline, Embase, the Cochrane Library, Google Scholar and relevant journals. Only studies published in English were included in the meta-analysis. We performed the last updated search on August 2018. The following text words and corresponding heading terms were used as search terms: “computed tomography”, “CT”, “bowel obstruction”, “small bowel obstruction”, “intestinal obstruction” and “small intestinal obstruction”. The MeSH terms and keywords used for the search are listed in **S2 Table**. Related articles and reference lists were manually searched to avoid omissions. After title screening, we evaluated abstracts for relevance and identified articles as included, excluded or requiring further assessment. At this stage, if a paper required further assessment, we contacted the study lead investigator by e-mail with a request for further information.

#### 2). Data extraction

The inclusion criteria were as follows: (a) original research focusing on the diagnostic value of CT in SBO; (b) CT as diagnostic index test; and (c) sufficient data available to calculate construct 2×2 contingency tables and corresponding 95% confidence interval (95% CI). The following exclusion criteria were used: (a) index test and reference standard were both used in CT; (b) reviews of the literature; (c) nonhuman studies; (d) not published in English. For studies reporting the same or overlapping data by the same authors, the most suitable studies with the largest number of cases or latest publication dates were selected. Two investigators (Z.L. and L.Z.) assessed each study independently and recorded eligibility, quality and outcomes. Disagreements regarding eligibility were noted in 5% of the articles (κ = 0.89), which were resolved by a third party through consensus. A third investigator (F.Y.) provided arbitration in case of disagreement.

We extracted the following study features: (a) study design and patient characteristics (i.e., first author, year of publication, country of origin, department of the first author, consecutive recruitment, number of patients, age, sex ratio, and inclusion criteria); (b) imaging techniques (i.e., CT type, collimation, slice thickness, and use of contrast agent); (c) image evaluation (i.e., number of readers, retrospective or prospective CT reading, consensus reading, and interests of diagnosis); (d) reference standard (i.e., time between admission and surgery, histopathologic analysis, surgery findings, and duration of the medical follow-up).

#### 3). Quantitative data synthesis

Independently and in duplicate, reviewers assessed the risk of bias using the tool of the second edition of the Quality Assessment of Diagnostic Accuracy Studies (QUADAS 2) [31] using Review Manager 5.3 (RevMan, The Cochrane Collaboration, Oxford, United Kingdom). The QUADAS 2 consisted of four key domains that discuss patient selection, index test, reference standard and flow of patients through the study and timing of the index tests and reference standard (flow and timing). Each domain was assessed in terms of the risk of bias, and the first three domains were also assessed in terms of concerns about applicability. For each included study, a description, a comment, and a judgment as “high”, “unclear”, or “low” risk of bias were provided for each of the domains. Studies with high risk of bias for any one or more key domains were considered to exhibit high risk of bias. Studies with low risk of bias for all key domains were considered to exhibit low risk of bias. Otherwise, studies were considered to exhibit an unclear risk of bias. We classified high risk of bias studies as low-quality studies, and the other studies were classified as high-quality studies.

The data of the two-by-two tables were used to calculate sensitivity and specificity of each study. We present individual study results graphically by plotting the estimates of sensitivity and specificity (and their 95% confidence interval [CI]) based on forest plots using bivariate random-effects and summary receiver operating characteristic curve models (SROC) [32, 33]. Heterogeneity was evaluated using the Cochrane Q test and the I^2^ statistic to assess the degree of inter-study variation. I^2^ values of 0 to 24.9%, 25 to 49.9%, 50 to 74.9%, and 75 to 100% were considered to indicate no, mild, moderate, and significant thresholds for statistical heterogeneity, respectively [34, 35]. Subgroup analyses were performed based on age (adult or pediatric), publication year (2000 as cutoff point), different imaging techniques (enhanced CT or not; slice thickness ≤5 mm or >5 mm) and different causes of SBO using meta-regression analysis.

We used the Deek funnel plot asymmetry test, which is the recommended tool for assessing risk of publication bias in meta-analyses of diagnostic test accuracy [36]. A P-value less than 0.10 for the slope coefficient indicated significant asymmetry. P-values less than or equal to 0.05 were considered indicative of a significant difference. Analyses were performed with “midas” modules in the Stata software (version 12.0; StataCorp, College Station, Tex).

## Results

### Eligible studies

**Fig 1:**
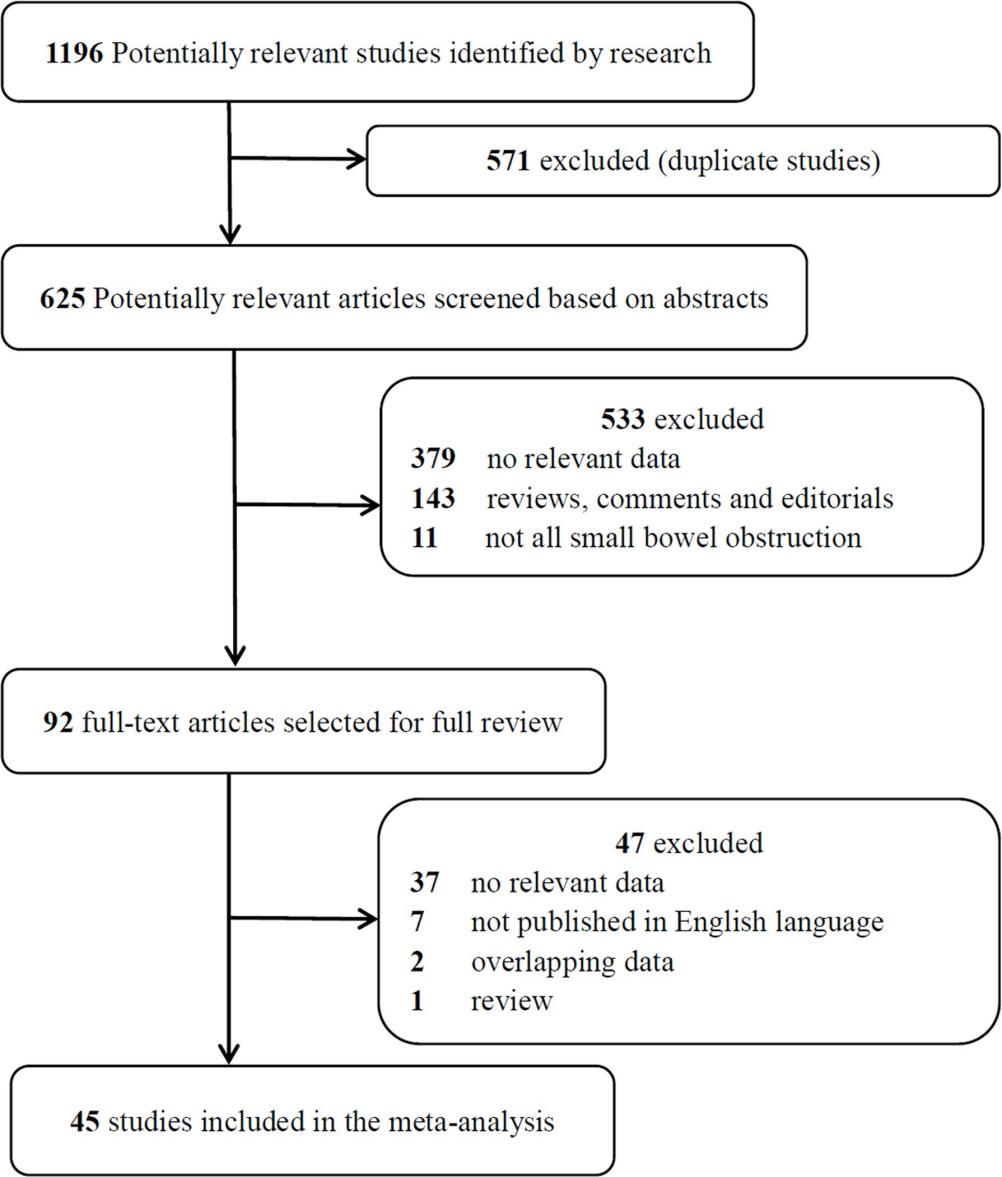
Flow chart of selection of studies.

The study selection process is presented in **Fig 1**. The literature search yielded 1196 potentially relevant records. We removed 571 duplicate studies after screening the titles. After evaluating the abstract of each study, 533 studies were excluded because they did not meet the inclusion criteria. Subsequently, we carefully read the full text of each of the remaining 92 studies and excluded 47 studies for the following reasons: no relevant data (n = 37), not published in English (n = 7), overlapping data (n = 2) and review (n = 1). Finally, 45 studies were included in the meta-analysis.

As shown in **Table 1**, the eligible studies were conducted from 1992 through 2018 with a total number of 4004 patients, and the sample size ranged from 20 to 256. All 45 studies focused on the diagnostic value of CT for SBO. Among them, 24 studies were from North America, 13 were from Asia and 8 were from Europe. A variety of outcomes were recorded in these studies, including diagnosis of SBO (n = 19), ischemia (n = 17), predicting surgical intervention (n = 7), etiology (n = 16) and transition point (n = 11).

**Table 1:** Key parameters extracted from the included studies.

Author, year	Counrty	Design	Adult or pediatric	No. of patients	mean age (y)	No. of male	Reference standard	Interests of diagnosis
Atri 2009[6]	Canada	Retrospective	adult	99	65	43	SF or CF	SBO, etiology, transition
Balthazar 1992[7]	USA	Retrospective	adult	19	63	7	SF	Ischemia
Balthazar 1997[8]	USA	Retrospective	adult	100	NR	NR	SF or CF	SBO, ischemia, etiology
Chang 2014[9]	Taiwan	Retrospective	adult	151	62	89	SF	PSI
Chang 2017[23]	Taiwan	Retrospective	Pediatric	31	7	NR	SF	Ischemia
Chuong 2016[24]	France	Retrospective	adult	158	71	61	SF or CF	Ischemia
Daneshmand 1999[10]	USA	Retrospective	adult	45	44	NR	SF or CF	SBO, etiology
Deshmukh 2011[11]	USA	Retrospective	adult	129	62	68	SF or CF	PSI
Donckier 1998[12]	Belgium	Retrospective	adult	54	62	30	SF or CF	Ischemia
Duda 2008[13]	USA	Retrospective	adult	194	50	NR	SF or CF	PSI
Filippone 2007[14]	Italy	Retrospective	adult	49	63	NR	SF	Etiology, transition
Frager 1994[16]	USA	Retrospective	adult	90	NR	NR	SF or CF	SBO, etiology, transition
Frager 1996[15]	USA	Retrospective	adult	60	NR	NR	SF	Ischemia
Fukuya 1992[17]	USA	Retrospective	adult	60	NR	NR	SF	SBO, etiology
Geffroy 2014[18]	France	Retrospective	adult	44	73	10	SF	Ischemia
Halepota 2018[47]	Pakistan	Retrospective	Pediatric	98	8	65	SF	SBO
He 2016[48]	China	Retrospective	adult	57	58	31	SF or DSA	Ischemia
Hwang 2009[27]	Korea	Prospective	adult	128	NR	NR	SF	PSI
Idris 2012[49]	Pakistan	Retrospective	adult	59	48	22	SF	Transition
Jabra 1997[50]	USA	Retrospective	Pediatric	20	9	NR	SF	Etiology
Jabra 2001[51]	USA	Retrospective	Pediatric	59	9	NR	SF	SBO
Jaffe 2006[52]	USA	Retrospective	adult	100	55	40	SF or CF	SBO
Jancelewicz 2009[53]	USA	Retrospective	adult	192	59	81	SF	Ischemia
Jang 2010[54]	Korea	Retrospective	adult	60	29	54	SF	Ischemia
Kato 2010[55]	Japan	Retrospective	adult	115	73	60	SF	Ischemia
Kim 2004[25]	Korea	Retrospective	adult	136	51	71	SF	Ischemia
Kulvatunyou 2015[56]	USA	Prospective	adult	202	60	100	SF	PSI
Maglinte 1993[57]	USA	Retrospective	adult	55	NR	NR	EC or SF	SBO
Maglinte 1996[38]	USA	Retrospective	adult	78	NR	NR	EC or SF	SBO
Makanjuola 1998[58]	Saudi Arabia	Retrospective	adult	49	32	27	SF or CF	SBO, ischemia, etiology
Matsushima 2016[59]	USA	Retrospective	adult	111	52	56	SF	PSI
Memon 2014[60]	Pakistan	Retrospective	adult	102	NR	NR	SF	Transition
Millet 2017[43]	France	Retrospective	adult	256	64	NR	SF	Ischemia
Obuz 2003[61]	Turkey	Retrospective	adult	41	NR	NR	SF or CF	SBO, ischemia, etiology
Peck 1999[19]	USA	Retrospective	adult	55	56	NR	SF or CF	SBO
Pongpornsup 2009[62]	Thailand	Retrospective	adult	35	56	25	SF or CF	SBO, etiology
Scrima 2017[63]	USA	Retrospective	adult	179	56	86	SF	PSI, transition
Shah 2008[64]	USA	Retrospective	adult	30	43	19	SF	Etiology, transition
Sheedy 2006[26]	USA	Retrospective	adult	61	67	25	SF	SBO, ischemia
Taourel 1995[65]	France	Prospective	adult	57	60	33	SF or CF	Etiology
Walsh 1998[20]	USA	Retrospective	adult	36	NR	NR	EC	SBO
Wang 2012[21]	Canada	Retrospective	Pediatric	47	10	32	SF	Etiology, transition
Xu 2013[66]	China	Retrospective	adult	94	NR	51	SF	SBO, etiology
Yaghmai 2006[22]	USA	Retrospective	adult	67	NR	NR	SF	SBO, etiology, transition
Zalcman 2000[41]	Belgium	Retrospective	adult	142	61	81	SF	Ischemia

Abbreviation: SF, surgical findings; CF, clinical findings, EC, enteroclysis; PSI, prediction of surgical intervention; DSA, digital subtraction angiography; NR, not reported.

### Assessment of methodological quality

**Fig 2:**
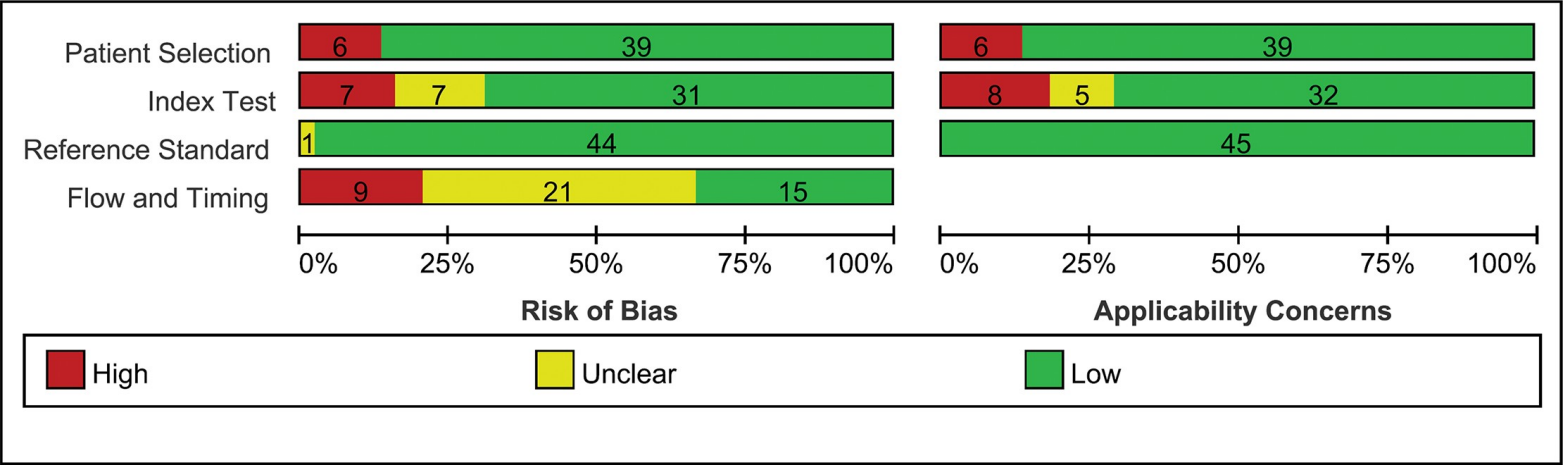
Quality assessment for risk of bias and applicability of included studies: QUADAS evaluation.

The summary and details of risk of bias were summarized in **Fig 2 and S1 File**. In summary, twelve (27%) studies that fulfilled all of the methodological criteria were judged to be at low risk of bias, and 20 (44%) exhibited high risk of bias. The remaining 13 (28%) were judged to be at unclear risk of bias. Among included studies, 6 (13%) studies had a high risk in the domain of patient selection given that the selection was based on specific or nonexhaustive causes of SBO. There was a high risk of bias with the index test in 7 (16%) studies that did not report information on CT performance parameters or reader number and expertise. In addition, most (93%) of included studies had a retrospective design, and 17 (38%) of these studies did not provide any information about whether the results of the reference standard were blinded to readers. Thus, the diagnostic performance of CT could have been overestimated. Only 15 (33%) studies reported an acceptable delay between index test and reference standard, and the other studies were considered to have a high risk or potential risk of flowing and timing bias due to unclear intervals. Meta-regression was performed to compare the different qualities of included studies.

### Value of CT in diagnosis of SBO

**Fig 3:**
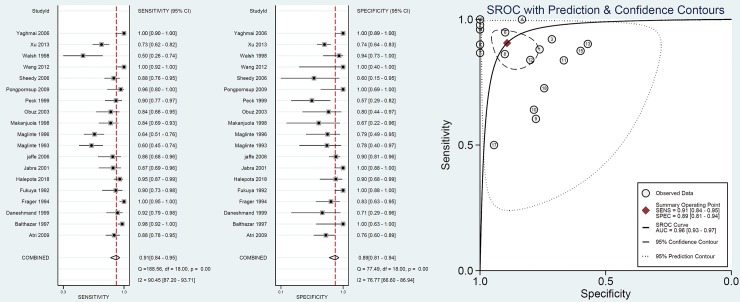
Value of CT in diagnosis of SBO.

Overall, nineteen studies including 1269 patients reported data on diagnostic performance of CT on SBO. As shown in **Fig 3**, summary analysis results showed the sensitivity and specificity of CT for SBO were 91% (95% CI: 84%, 95%) and 89% (95% CI: 81%, 94%), respectively, and there significant heterogeneity (I^2^>75%) was noted. The SROC demonstrated an area under the curve of 0.96 (95% CI: 0.93, 0.97). Subgroup analysis results using meta-regression are presented in **Table 2**. There was no difference in diagnostic value of CT for SBO between adult and pediatric patients (sensitivity 89% vs. 96%, P = 0.12; specificity 86% vs. 90%, P = 0.08). Unenhanced CT was associated with a similar diagnostic value for SBO compared with enhanced CT. In addition, similar results were found between different slice thicknesses (≤5 mm or >5 mm), different study qualities (high or low quality) or publication year (<2000 or ≥2000).

**Table 2:** Pooled estimates of the diagnostic performance of CT in SBO and SBO with ischemia.

**Variable**	**No. of studies**	**Pooled Sensitivity (95% CI)**	**P value**	**Pooled Specificity (95% Cl)**	**P value**
**Diagnosis for SBO**					
**Over all**	19	91% (84%, 95%)		89% (81%, 94%)	
**Age**			0.12		0.08
Adult	16	89% (83%, 95%)		86% (79%, 93%)	
Pediatric	3	96% (90%, 100%)		90% (84%, 97%)	
**Enhanced CT or not**			0.39		0.74
Enhanced	16	90% (84%, 96%)		90% (84%, 97%)	
Unenhanced	3	93% (83%, 100%)		82% (62%, 100%)	
**Publication year**			0.56		0.61
<2000	9	88% (78%, 97%)		86% (75%, 97%)	
≥2000	10	93% (87%, 99%)		82% (62%, 100%)	
**Slice thickness**			0.66		0.26
≤5 mm	8	93% (87%, 100%)		89% (79%, 99%)	
> 5mm	11	88% (78%, 97%)		86% (75%, 97%)	
**Quality of studies**			0.81		0.87
High	13	93% (89%, 98%)		91% (84%, 98%)	
Low	6	81% (66%, 95%)		83% (70%, 97%)	
**Diagnosis for ischemia**					
**Over all**	17	82% (67%, 91%)		92% (86%, 95%)	
**Enhanced CT or not**					
Enhanced	16	83% (71%, 94%)		92% (87%, 96%)	
Unenhanced	1	64%		93%	
**Publication year**			0.21		0.09
<2000	5	91% (78%, 100%)		93% (86%, 100%)	
≥2000	12	77% (62%, 93%)		91% (86%, 96%)	
**Etiology**			0.03		0.10
Adhesion	2	96% (89%, 100%)		85% (68%, 100%)	
Routine causes	15	78% (65%, 91%)		92% (88%, 96%)	
**Quality of studies**			0.79		0.27
High	12	83% (71%, 96%)		92% (88%, 97%)	
Low	5	76% (50%, 100%)		89% (80%, 98%)	

### Value of CT in diagnosis of ischemia

**Fig 4:**
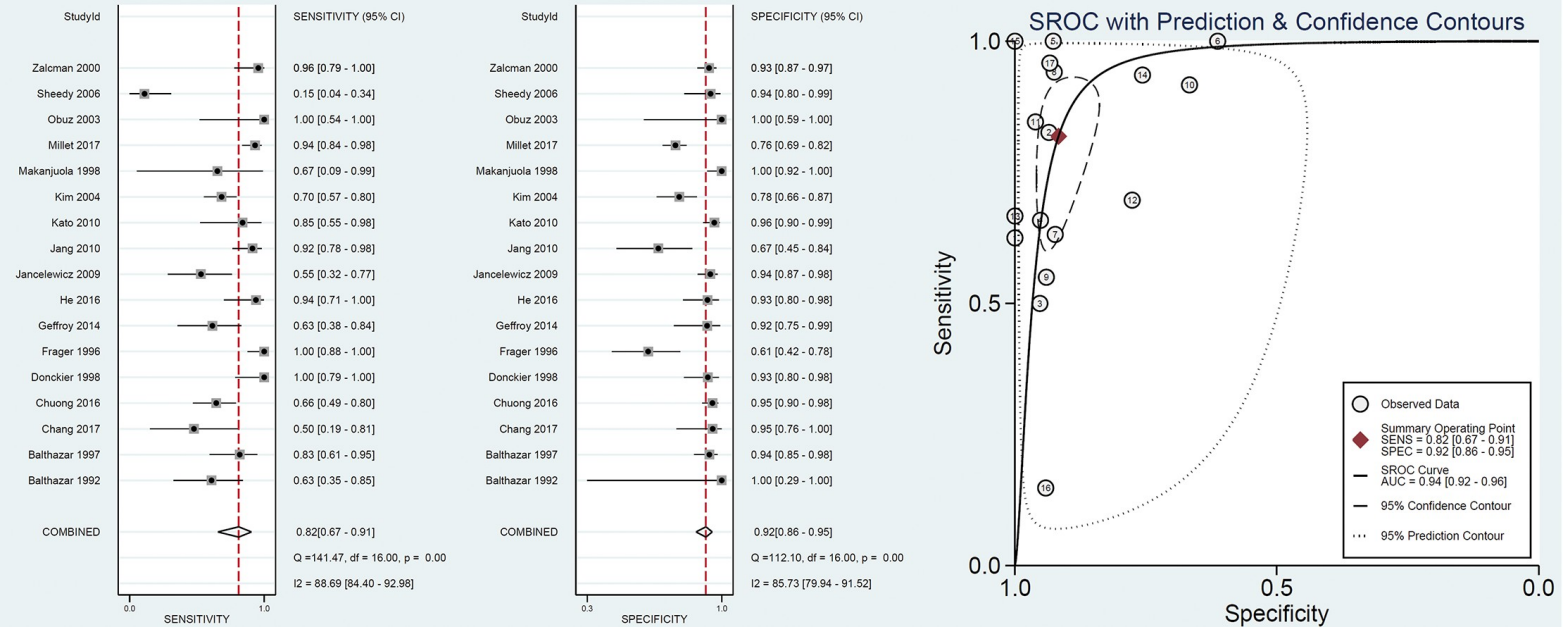
Value of CT in diagnosis of ischemia.

Seventeen studies (including 1575 patients) reported the value of CT in diagnosis of SBO with ischemia. The pooled sensitivity and specificity were 82% (95% CI: 67%, 91%) and 92% (95% CI: 86%, 95%), respectively, and there was significant heterogeneity (I^2^>75%) (**Fig 4**). The SROC demonstrated an area under the curve of 0.94 (95% CI: 0.92, 0.96). Only one study (18) reported the value of unenhanced CT in diagnosis of ischemia and the sensitivity and specificity were 64% and 93%. In subgroup analysis (**Table 2**), no difference was found between the enhanced and the unenhanced CT (sensitivity 83% vs. 64%, P = 0.23; specificity 92% vs. 93%, P = 0.79). However, higher sensitivity was found in adhesive SBO compared with routine causes (96% vs. 78%, P = 0.03), but the specificity was not significant (85% vs. 92%, P = 0.1). In addition, no difference was found between different study qualities (high or low quality) or publication year (<2000 or ≥2000).

### Value of CT in predicting surgical intervention

**Fig 5:**
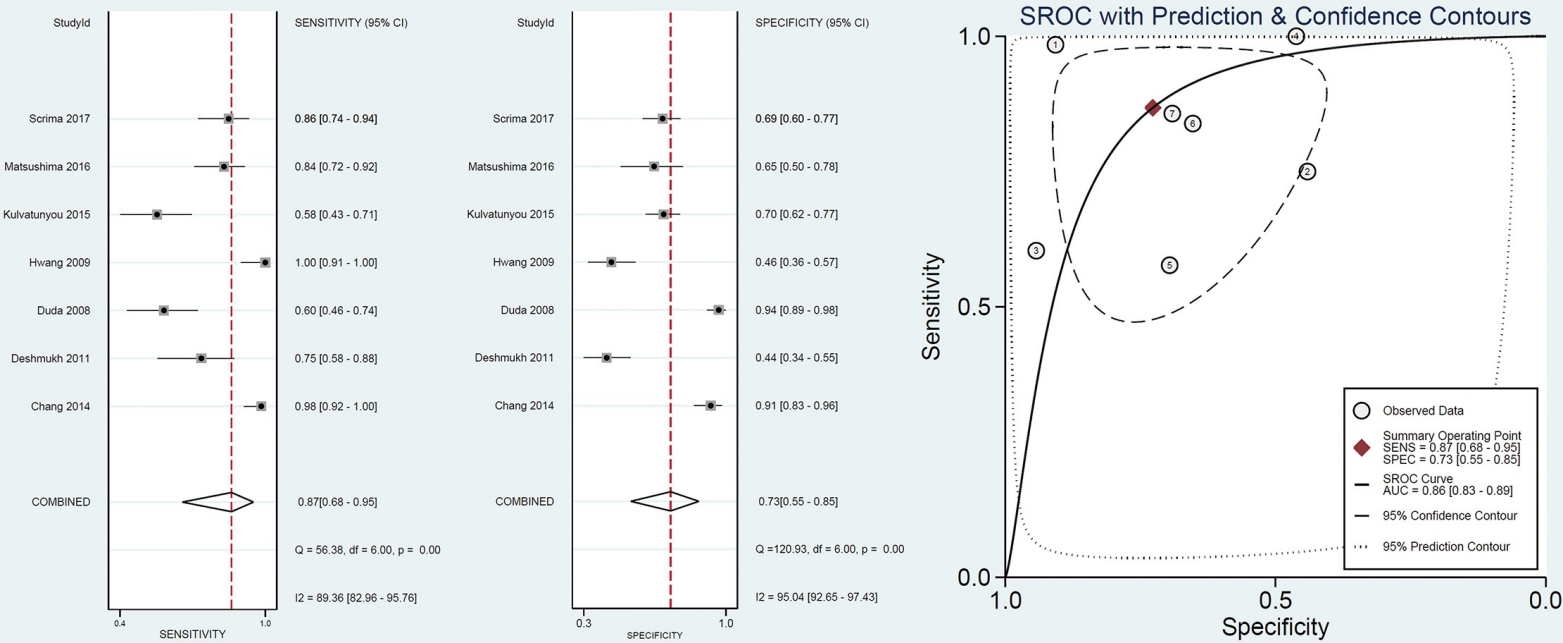
Value of CT in predicting surgical intervention.

As shown in **Fig 5,** seven studies including 1094 patients presented the value of CT in predicting surgical intervention. Among them, 3 studies focused on adhesive SBO, and the other 4 studies focused on SBO with routine causes. The results of the summary analysis showed the sensitivity and specificity were 87% (95% CI: 68%, 95%) and 73% (95% CI: 55%, 85%), respectively, and there was significant heterogeneity (I^2^>75%). In subgroup analysis, higher sensitivity was found in adhesive SBO compared with routine causes (96% vs. 71%, P = 0.05), but the specificity was not significant (71% vs. 74%, P = 0.7).

### Value of CT in etiology

Sixteen studies (including 961 patients) reported the value of CT in SBO etiology. The pooled sensitivity was 83% (95% CI: 76%, 88%), and there was significant heterogeneity (I^2^>75%). Subgroup analysis results using meta-regression were presented in **Table 3**. The pooled sensitivity values of CT for adhesion, hernia and tumor were 95% (I^2^ = 52%), 70% (I^2^ = 57%) and 82% (I^2^ = 37%), respectively.

**Table 3:** Sensitivity of CT in diagnosis of etiology in SBO.

	**No. of studies**	**Sensitivity (95% CI)**	**I**^**2**^
Over all	16	83% (76%, 88%)	77%
Adhesion	10	95% (89%, 98%)	52%
Hernia	9	70% (44%, 88%)	57%
Tumor	9	82% (72%, 89%)	33%

### Value of CT in transition point diagnosis

Overall, eleven studies including 823 patients presented the value of CT in diagnosis of transition point of SBO. Summary analysis results showed that the sensitivity and specificity were 92% (95% CI: 87%, 95%) and 87% (95% CI: 74%, 95%), respectively, and significant heterogeneity was noted (I^2^>75%).

### Publication bias

**Fig 6:**
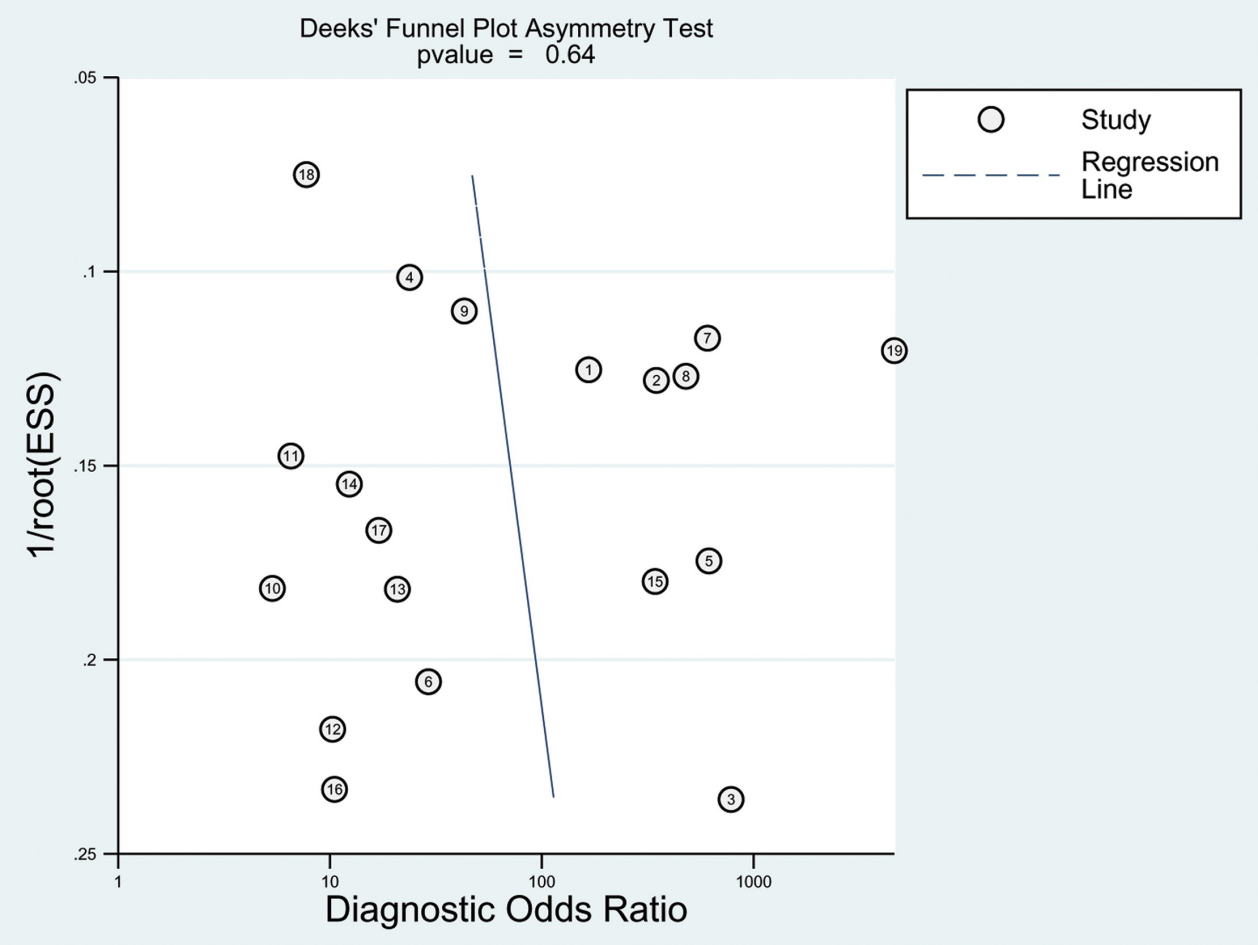
Illustration of publication bias.

The impact of publication bias on meta-analysis results was assessed using Deeks’ funnel plots. The shapes of the funnel plots for the pooled sensitivity and specificity of CT performance for SBO revealed obvious symmetry (P = 0.64), indicating that the meta-analysis was not affected by publication bias (**Fig 6**).

## Discussion

We performed a systematic review of the literature and identified 45 studies (more than 4000 patients) reporting the diagnostic performance of CT for SBO. The results of this study showed that CT should be the choice for the overall diagnosis of SBO, which has considerable accuracy in diagnosis of SBO, ischemia, predicting surgical intervention, etiology and transition point. Similar diagnostic value of CT for SBO was found between adult and pediatric patients. We also found that unenhanced CT had similar power in diagnosis of SBO compared with enhanced CT. In addition, CT was associated with higher sensitivity for ischemia in adhesive SBO compared with routine causes of SBO, but no difference in specificity was noted. Furthermore, regarding etiology, CT had significant sensitivity for adhesion identification.

Currently, plain film radiography (X-ray), CT, ultrasound and magnetic resonance imaging (MRI) are utilized as diagnostic modalities for suspected SBO. Due to easier accessibility and lower cost, plain film radiography (X-ray) is typically the initial imaging choice for the evaluation of SBO. However, this imaging modality is often nondiagnostic with poor sensitivity (46–69%) and specificity (57–67%) [37, 38]. In contrast, our study reported significant sensitivity and specificity of CT for SBO at 91% and 89%, respectively. Ultrasound also represents a convenient and inexpensive diagnostic tool that could be performed at bedside, especially in the emergency department. A recent meta-analysis identified 11 studies and reported a high sensitivity (92%) and specificity (97%) of ultrasound for SBO [39]. However, limited evidence is available for the use of ultrasound for diagnosis of small bowel ischemia, which is an important complication of SBO and an indication for emergency surgical intervention [40]. Thus, CT, rather than X-ray or ultrasound, was recommended as the first choice in the overall diagnosis of SBO [67, 68].

As reported in our study, CT played a key role in the diagnosis of SBO and the diagnosis of small bowel ischemia. Many CT findings, such as decreased enhanced bowel wall, wall thickness, mesenteric congestion, mesenteric fluid, and peritoneal fluid, had been reported as findings related to bowel ischemia [8, 23, 24, 26, 41]. A previous meta-analysis [42] including nine studies reported that a hypoenhanced bowel wall (specificity 95%) was highly predictive of ischemia, and the absence of mesenteric fluid (sensitivity 89%) was a reliable finding to exclude strangulation. A recent study including 256 patients with adhesive SBO found that peritoneal fluid (sensitivity 89%) instead of mesenteric fluid (sensitivity 73%) had the highest sensitivity among CT findings, and hypoenhanced bowel wall, free peritoneal gas, pneumatosis or venous gas were all associated with high specificity (96–99%) [43].

Several studies [6, 18, 24] focused on the diagnostic value of unenhanced CT for SBO, which might be more safer for the patients with the high risk of contrast agent-induced nephropathy [69]. Atri et al [6] reported that unenhanced CT had similar accuracy to diagnose mechanical small bowel obstruction compared with enhanced CT. For ischemia, a retrospective study [18] showed that increased bowel-wall attenuation on unenhanced images had 100% specificity and 56% sensitivity. Furthermore, a recent study [24] found that the addition of unenhanced CT to contrast-enhanced CT could improve the sensitivity, diagnostic confidence, and observer agreement for the diagnosis of ischemia. However, due to the few evidence, the effect of unenhanced CT for ischemia should be reconfirmed in the future. Although similar diagnostic power between unenhanced and enhanced CT was found for SBO in this review, enhanced CT might be more powerful in the diagnosis of ischemia, etiology and predicting surgical intervention [67, 68].

In addition, a few studies [44–46] reported the diagnostic value of MRI for patients with suspected SBO, especially for pregnant women. A small observational study [44] reported that cine MRI was a feasible and promising technique for diagnosing strangulation of SBO with high sensitivity (100%) and specificity (93%). However, compared with CT, MRI might not be convenient (especially at night), has a longer scan time, and might not be as reliable in identifying the etiology of SBO [67].

This study first comprehensively evaluated the diagnostic utility of CT for SBO, including multiple relevant outcomes, such as diagnostic accuracy, ischemia, predicting surgical intervention, etiology and transition point. This study included data from more than 4,000 patients, 45 studies, and 13 countries from different regions of North America, Asia and Europe. Two independent investigators also rigorously assessed its methodological quality.

However, this systematic review and meta-analysis has several limitations. First, most (93.3%) of included studies were retrospective studies, which may overestimate the diagnostic value of CT, thus, high-quality prospective studies are needed in the future. Second, 66% of included studies are considered to exhibit a high risk or potential risk of flowing and timing bias due to an unclear interval between index test and reference standard with significant heterogeneities of primary outcomes. However, similar outcomes was found in the subgroup analysis of high-quality included studies. Third, data are limited with respect to the pediatric population, limiting applicability to this subgroup. Fourth, the literature on the use of unenhanced CT for ischemia is also limited (only one study included), highlighting the need for future investigations on this topic. Finally, although no publication bias was found in this meta-analysis, only published studies with selective databases were included for analysis, and the unavailability of unreported outcomes could have resulted in reporting bias. Regardless of these limitations, we sought to minimize bias throughout our study by using strict method identification, data selection, and statistical analysis, as well as controlling for publication bias and performing sensitivity analyses.

## Conclusion

This meta-analysis suggests that CT has considerable accuracy in diagnosis of SBO, ischemia, predicting surgical intervention, etiology and transition point. Further large-sample, high-quality prospective studies focusing on the performance of unenhanced CT for SBO with ischemia are needed.

## Supporting information

S1 Table PRISMA checklist. (DOCX)

S2 Table Characteristics of the computed-assisted literature search strategy. (DOCX)

S1 File Methodological criteria of the included studies: QUADAS evaluation. (TIF)
