# Supplementary material for: Diagnostic utility of CT for small bowel obstruction: Systematic review and meta-analysis
Source: PLoS One. 2019 Dec 30;14(12):e0226740. doi: 10.1371/journal.pone.0226740 (PMC6936825; doi:10.1371/journal.pone.0226740)
Supplement: S2 Table — (DOCX) [file pone.0226740.s002.docx]

S2 Table. Characteristics of the computed-assisted literature search strategy

Pubmed

1. "intestinal obstruction"[MeSH Terms]

2. "intestinal"[All Fields] AND "obstruction"[All Fields]

3. "small intestinal obstruction"[All Fields]

4. "small bowel obstruction"[All Fields]

5. "bowel"[All Fields] AND "obstruction"[All Fields]

6. #1 OR #2 OR #3 OR #4 OR #5

7. "ct"[All Fields]

8. "tomography"[All Fields] AND "x-ray"[All Fields] AND "computed"[All Fields]

9. "x-ray computed tomography"[All Fields]

10. "computed"[All Fields] AND "tomography"[All Fields]

11. "computed tomography"[All Fields]

12. "tomography, x-ray computed"[MeSH Terms]

13. #7 OR #8 OR #9 OR #10 OR #11 OR #12

14. #6 AND #13
