# Supplementary figures and images for: Diagnostic utility of CT for small bowel obstruction: Systematic review and meta-analysis
Source: PLoS One. 2019 Dec 30;14(12):e0226740. doi: 10.1371/journal.pone.0226740 (PMC6936825; doi:10.1371/journal.pone.0226740)

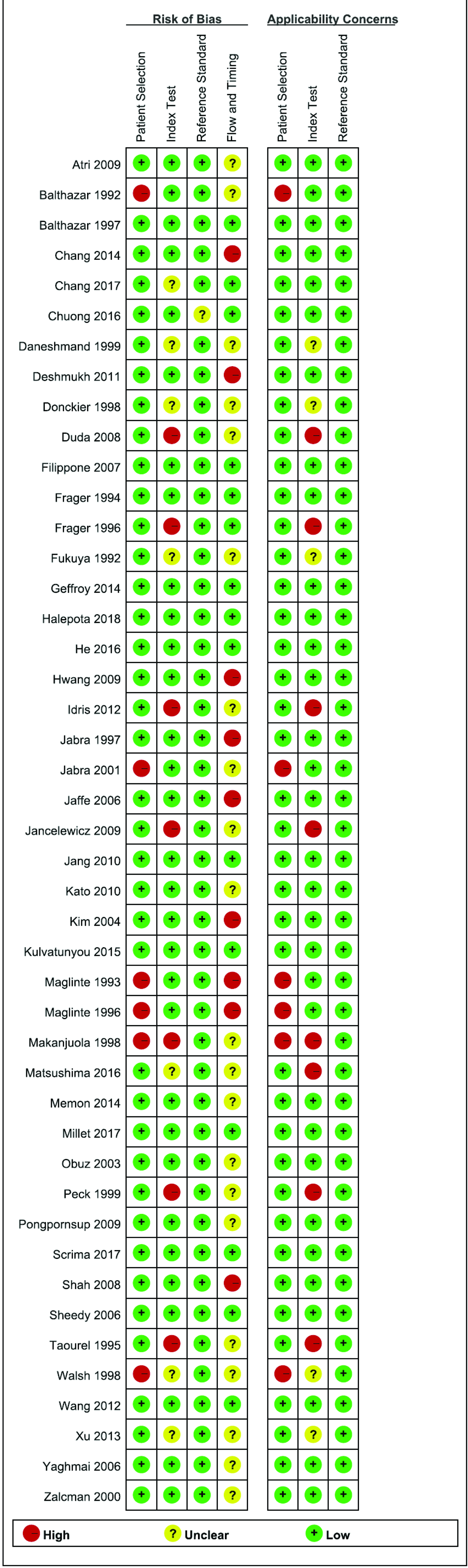

Supplement: S1 File — (TIF) [file pone.0226740.s003.tif]
